# Errata

**DOI:** 10.1590/1984-3143-AR2019-0068

**Published:** 2020-07-01

In the article “Improvement of *in vitro* fertilization by a tannin rich vegetal extract addition to frozen thawed boar sperm”, published in journal Animal Reproduction, 2020, volume 17, issue 2, the DOI number was published incorrectly.

Where it reads:

https://doi.org/10.21451/1984-3143-AR2019-0130

It should be read:

https://doi.org/10.1590/1984-3143-AR2019-0130

In the article “Characterization of mesenchymal stem cells derived from adipose tissue of a cougar (*Puma concolor*)”, published in journal Animal Reproduction, 2020, volume 17, issue 2, the DOI number was published incorrectly.

Where it reads:

https://doi.org/10.21451/1984-3143-AR2019-0109

It should be read:

https://doi.org/10.1590/1984-3143-AR2019-0109

